# Serotonin Augments Gut Pacemaker Activity via 5-HT_3_ Receptors

**DOI:** 10.1371/journal.pone.0024928

**Published:** 2011-09-15

**Authors:** Hong-Nian Liu, Susumu Ohya, Yuji Nishizawa, Kenta Sawamura, Satoshi Iino, Mohsin Md Syed, Kazunori Goto, Yuji Imaizumi, Shinsuke Nakayama

**Affiliations:** 1 Department of Cell Physiology, Nagoya University Graduate School of Medicine, Nagoya, Japan; 2 Department of Anatomy and Cell Biology, Nagoya University Graduate School of Medicine, Nagoya, Japan; 3 Department of Molecular and Cellular Pharmacology, Graduate School of Pharmaceutical Sciences, Nagoya City University, Nagoya, Japan; 4 Department of Anatomy, Faculty of Medical Sciences, University of Fukui, Fukui, Japan; Charité, Campus Benjamin Franklin, Germany

## Abstract

Serotonin (5-hydroxytryptamine: 5-HT) affects numerous functions in the gut, such as secretion, muscle contraction, and enteric nervous activity, and therefore to clarify details of 5-HT's actions leads to good therapeutic strategies for gut functional disorders. The role of interstitial cells of Cajal (ICC), as pacemaker cells, has been recognised relatively recently. We thus investigated 5-HT actions on ICC pacemaker activity. Muscle preparations with myenteric plexus were isolated from the murine ileum. Spatio-temporal measurements of intracellular Ca^2+^ and electric activities in ICC were performed by employing fluorescent Ca^2+^ imaging and microelectrode array (MEA) systems, respectively. Dihydropyridine (DHP) Ca^2+^ antagonists and tetrodotoxin (TTX) were applied to suppress smooth muscle and nerve activities, respectively. 5-HT significantly enhanced spontaneous Ca^2+^ oscillations that are considered to underlie electric pacemaker activity in ICC. LY-278584, a 5-HT_3_ receptor antagonist suppressed spontaneous Ca^2+^ activity in ICC, while 2-methylserotonin (2-Me-5-HT), a 5-HT_3_ receptor agonist, restored it. GR113808, a selective antagonist for 5-HT_4_, and O-methyl-5-HT (O-Me-5-HT), a non-selective 5-HT receptor agonist lacking affinity for 5-HT_3_ receptors, had little effect on ICC Ca^2+^ activity. In MEA measurements of ICC electric activity, 5-HT and 2-Me-5-HT caused excitatory effects. RT-PCR and immunostaining confirmed expression of 5-HT_3_ receptors in ICC. The results indicate that 5-HT augments ICC pacemaker activity via 5-HT_3_ receptors. ICC appear to be a promising target for treatment of functional motility disorders of the gut, for example, irritable bowel syndrome.

## Introduction

Special interstitial cells with abundant c-Kit receptors on their surface are distributed throughout the gastrointestinal tract. These cells are referred to as interstitial cells of Cajal (ICC) due to the histological features of the network [1]–[3]. It is now considered that ICC in the myenteric region act as pacemaker cells, and produce gut movements in concert with enteric neurones and smooth muscle cells [4]–[6]. Numerous neurotransmitters and hormones are likely to affect ICC activity, and thereby modulate gut motility.

Serotonin (5-hydroxytryptamine: 5-HT), well known for mood control in the brain, also plays a crucial role in cellular signalling in the gut. Indeed, enterochromaffin cells release the majority (>90%) of 5-HT in the human body in response to the pressure of intraluminal content and other noxious stimuli [7]. Some enteric neurones in the descending peristaltic reflex pathway also release 5-HT as a neurotransmitter [8]–[10]. Since enteric neurones and smooth muscle cells express various 5-HT receptors depending upon cell type and location of the cell, and their functions are critically affected by this signalling molecule [11], [12], 5-HT receptors are key targets in pharmacological interventions of gut functional disorders, as well as psychiatric disorders of the brain.

It is thought that oscillations of the intracellular (cytosolic) Ca^2+^ concentration ([Ca^2+^]_i_) in ICC cells underlie gut pacemaker activity. Namely, periodic activation of Ca^2+^-sensitive ion channels in the plasma membrane generates pacemaker potentials [13], [14]. Previously, we demonstrated that spontaneous electrical activity occurs in synchrony with [Ca^2+^]_i_ oscillations in ICC, and that coordinated actions of intracellular Ca^2+^ release channels and transmembrane Ca^2+^ influx pathways underlie ICC [Ca^2+^]_i_ oscillations [15]–[17].

In the present study, we provide evidence that 5-HT regulates ICC pacemaker activity. We performed Ca^2+^ imaging and potential mapping of ICC pacemaker activity using fluorescent Ca^2+^ probes and microelectrode array (MEA), respectively, and found that 5-HT enhances both Ca^2+^ and electric activities of ICC via 5-HT_3_ receptors, which are nonselective cation channels permeable to Ca^2+^. We also carried out RT-PCR and immunostaining to confirm the expression of 5-HT_3_ receptors in ICC. Our findings suggest that 5-HT modulation of ICC activity should also be considered for gut motility disorders, for example, irritable bowel syndrome with a prevalence of around 10% [18]. Interestingly, this disease is known to be frequently complicated by psychiatric illness and mood disorders.

## Materials and Methods

### Animals

Animals used in the present study were treated ethically. All procedures were approved by the Institutional Animal Care and Use Committee. BALB/c (wild-type) and *W/W^v^* mice (3–6 weeks after birth) were killed by cervical dislocation, after being anaesthetized with diethyl ether. Unless otherwise stated, BALB/c mice were used in all experiments.

### Ca^2+^ imaging

Cell cluster preparations were used in Ca^2+^ imaging [16], [19]. Although we detected 5-HT-augmentation of ICC Ca^2+^ activity in isolated ileal musculature segments containing the myenteric plexus (Figure S1; Video S1 and Video S2), we used cell cluster preparations in Ca^2+^ imaging to examine numerous drugs related to 5-HT, because it was difficult to stably load Ca^2+^ indicators in many intact muscle segments.

The musculature along with the myenteric plexus were carefully dissected from the ileum, and incubated in Ca^2+^-free Hanks' solution containing collagenase (1 mg/ml, Wako Chemical, Osaka, Japan), trypsin inhibitor (2 mg/ml, type I-S, Sigma, St Louis, MO, USA), ATP (0.3 mg/ml, Seikagakukogyo, Tokyo, Japan), and bovine serum albumin (2 mg/ml, Sigma) for 40 min at 37°C. The musculature preparation was then triturated with fire-blunted glass pipettes. The resultant cell clusters were plated onto a lab-made culture dish, and kept in Dulbecco's modified Eagle's medium (Sigma) supplemented with 10% foetal bovine serum (Sigma) and antibiotics (30 µg/ml streptomycin and 30 units/ml penicillin; Sigma) at 37°C for 2–3 days.

The cultured cell cluster preparations were incubated in ‘normal’ solution containing approximately 8 µM Fluo3-AM (acetoxymethly ester of Fluo-3: Dojindo, Kumamoto, Japan) and detergents (0.02% Pluronic F-127: Dojindo; 0.02% cremophor EL, Sigma) for 3–4 h at room temperature. A CCD camera system (Argus HiSCA, Hamamatsu Photonics, Hamamatsu, Japan) was used to continuously monitor digital images of Fluo-3 emission light. The cell clusters were illuminated at 488 nm and emission light of 515–565 nm was detected. The temperature of the recording chamber was kept at 35°C using a micro-warm plate system (MP10DM, Kitazato Supply, Fujinomiya, Japan). Digital images (0.963 µm/pixel) were normally collected at 300 ms intervals. Changes in fluorescence emission intensity (*F*) were expressed as *F*_t_ */F*_0_, where *F*_0_ is the basal fluorescence intensity. Ratio-images were constructed by dividing each Ca^2+^ image with a Ca^2+^ image acquired at a basal [Ca^2+^]_i_ time after subtracting background fluorescence. The frequency of spontaneous [Ca^2+^]_i_ oscillations in the presence of nifeipine (and TTX) did not differ from that of spontaneous oscillatory inward currents previously measured by the patch clamp technique [20]. We thus judged that the procedure for loading fluo-3 was appropriate, and the spontaneous [Ca^2+^]_i_ activity reflected pacemaker activity in ICC. This notion also agrees well with previous reports in which dihydropyridine (DHP) Ca^2+^ antagonists selectively suppress [Ca^2+^]_i_ activity in smooth muscle by blocking L-type Ca^2+^ channels [13], [19].

**Figure 1:**
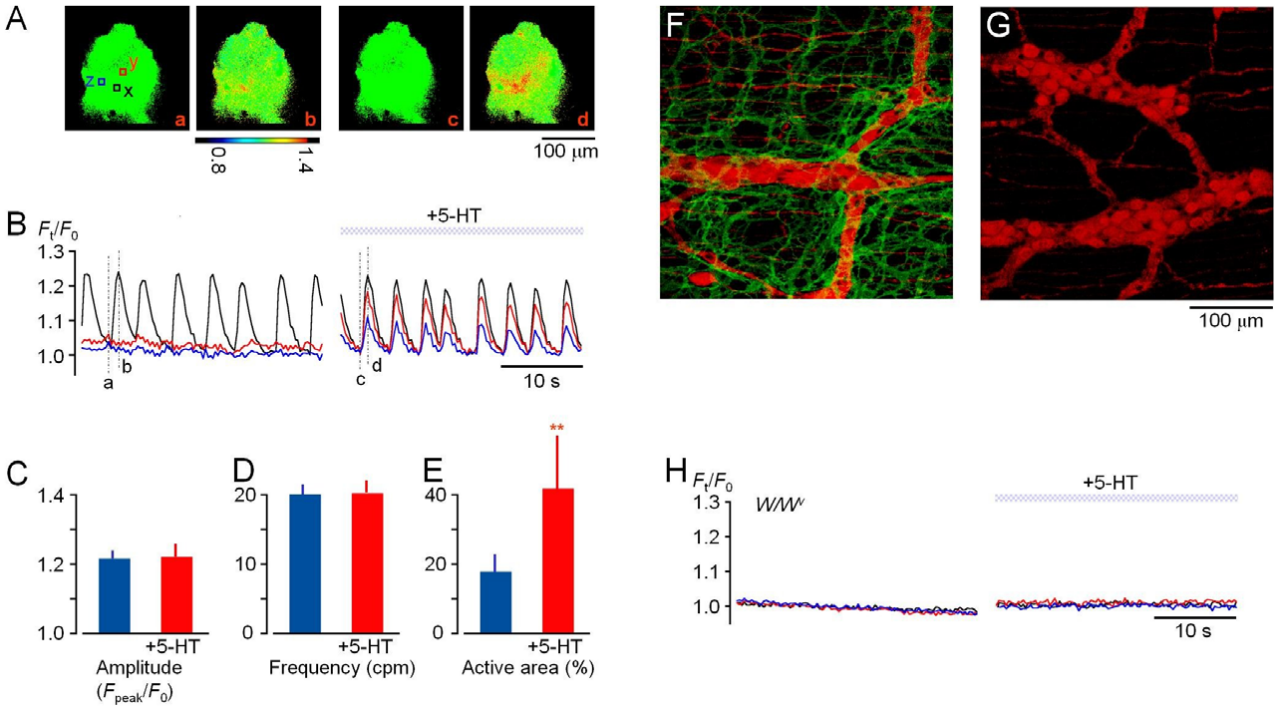
5-HT potentiates ICC pacemaker [Ca^2**+**^]_i_ activity. A) Ca^2+^ images acquired from a cell cluster preparation in control (a-b) and 5 min after 5-HT (10 µM) application (c-d). B) Time course of pacemaker [Ca^2+^]_i_ activity recorded in the three squares (x, y and z) indicated in Ba. Dotted lines correspond to the times when images (Ba-d) were acquired. Changes in fluorescence emission intensity (*F*) are expressed as *F*_t_ */F*_0_, where *F*_0_ is the basal fluorescence intensity. C-E) Bars show changes in the peak amplitude (C), frequency (D), and active area of spontaneous pacemaker [Ca^2+^]_i_ activity (E) (Mean ± s.d., n = 22). Asterisks, *P*<0.05 compared to control. F-G) Immunohistochemistry of the ileal myentric plexus (MyP) layer in wild-type (F) and *W/W^v^* mice (G) lacking ICC in this region of the small intestine. Red and green represent neurones (PGP9.5) and ICC (c-Kit), respectively. H) The lack of effect of 5-HT (10 µM) in a cell cluster preparation from *W/W^v^* mice.

**Figure 2:**
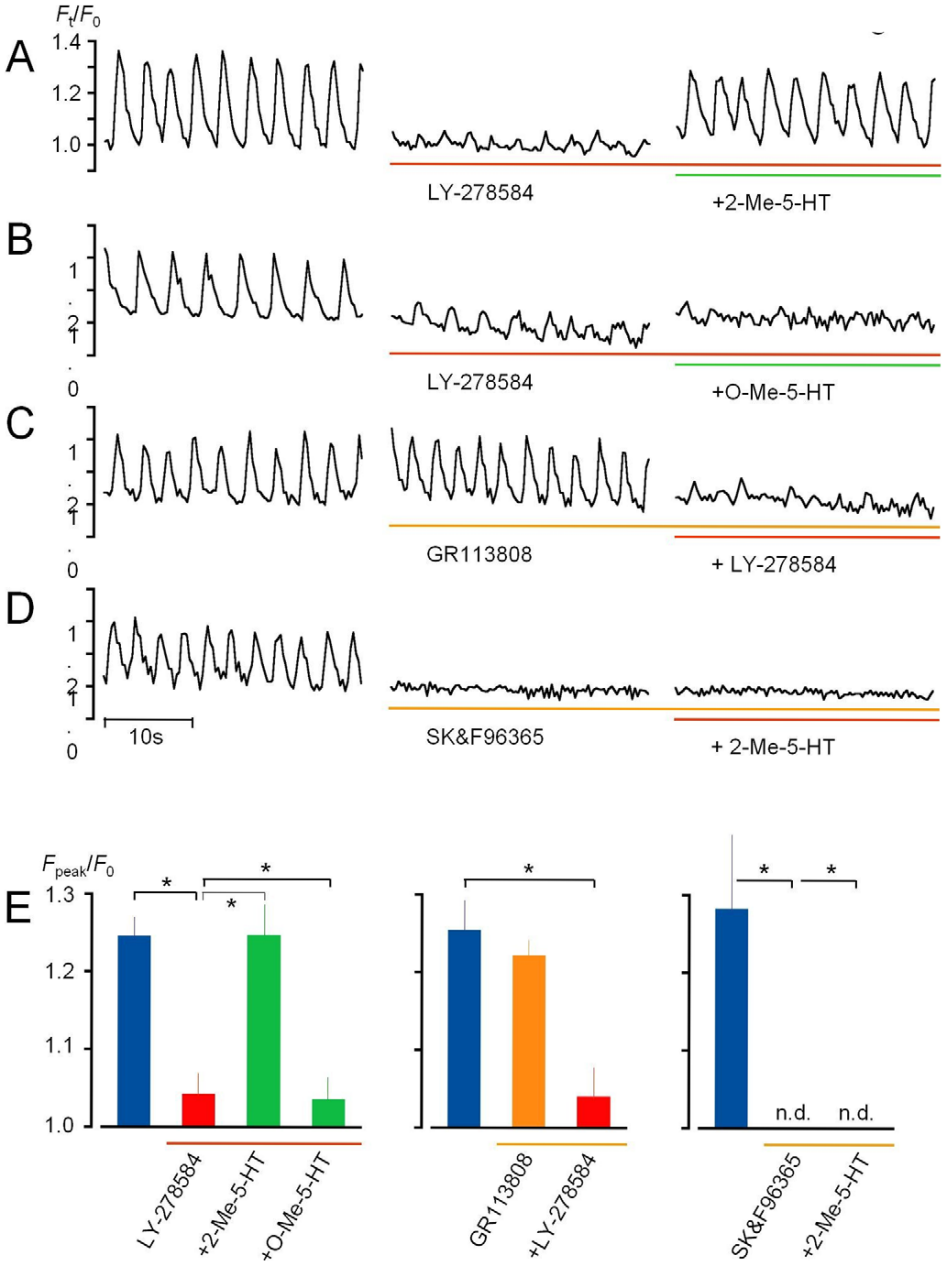
Evidence that endogenous 5-HT generates basal pacemaker [Ca^2**+**^]_i_ activity via 5-HT_3_. A-D) Examples of effects of drugs relating to 5-HT. Each time course of changes in [Ca^2+^]_i_ activity was acquired 3–5 min after application of drugs. All measurements were carried out in the presence of 1 µM nifedipine. LY-278584, 2-Me-5-HT, GR113808 and O-Me-5-HT were applied at 10 µM, and SK&F96365 was applied at 40 µM. E) Graphs summarizing the effects of drugs relating to 5-HT on the amplitude of pacemaker [Ca^2+^]_i_ oscillations. Blue bar represents the control. Blue and red bars in the left graph include both experiments (n = 10) for control and subsequent application of LY-278584 shown in A (n = 5) and B (n = 5). Statistical significance of the effects of 2-Me-5-HT and O-Me-5-HT were evaluated by comparing with each control observed in the presence of LY-278584 (n = 5, each). Middle and right graphs represent experiments shown in C (n = 5) and D (n = 5), respectively. Asterisks (*P*<0.05). n.d. (No [Ca^2+^]_i_ activity detected.).

In preliminary experiments, we checked the effects of several concentrations of 5-HT receptor agonists and antagonists in order to assess which subtypes of 5-HT receptors are responsible for the augmentation of [Ca^2+^]_i_ oscillations in ICC. First, 5-HT was examined at 1, 3, 10 and 50 µM (n = 3–4). The active area of [Ca^2+^]_i_ oscillations was nearly the same (102% of the control) at 1–3 µM, and increased to ∼145% at 10 µM and to ∼149% at 50 µM. We thus compared the effects of 5-HT and other 5-HT receptor agonists at 10 µM (Fig. 1 and 2). LY-278584, a 5-HT_3_ antagonist, did not significantly suppress [Ca^2+^]_i_ oscillations below 10 µM in 10 min. In order to minimize the deterioration of fluo-3 fluorescence during illumination, 10 µM of LY-278584 was normally applied (Fig. 2A–B). Also, according to previous experiments in enteric neurones and ICC, 10 µM GR113808 [21] and 40 µM SK&F96365 [17] were used to inhibit 5-HT_4_ receptors and Ca^2+^-permeable transmembrane channels, respectively (Fig. 2C–D).

### Electrical recording

An 8×8 planar microelectrode array (150 µm in polar distance) connected to a 64-channel amplifier (MED 64 System: Alpha Med Science, Osaka, Japan) was used to simultaneously record electrical field potentials of ∼1 mm^2^ square [22], [23]. Ileal musculature segments (∼5 mm×20 mm) containing the myenteric plexus were fixed using a brain slice anchor (SDH series, Harvard Apparatus Japan, Tokyo, Japan) in the recording chamber kept at 35°C on a heater, and were superfused with ‘normal’ extracellular solution at a constant rate of 2 ml/min. The extracellular solution contained nifedipine (Sigma) and TTX (LKT Laboratories: St Paul, MN, USA) in order to isolate ICC pacemaker activity by suppressing smooth muscle and neural activities. Slow electrical potentials were recorded by applying a high-pass filter of 0.1 Hz to stabilize the baseline drift [24]. A sampling rate of 20 kHz was applied.

In field potential data processing, the digital resolution was reduced to 50 ms (20 Hz) by thinning out the recording points at 1∶1000, and an FFT-based digital band-pass filter (0.04–0.5 Hz) was additionally applied. The effects of 5-HT and 2-Me-5-HT on ICC pacemaker electrical activity were evaluated using a power spectrum (9.4–27.0 cpm). Two-dimensional field potential images were constructed by calculating the values at the desired location via spline interpolation (with 50 points between each electrode), using the MATLAB software package (Mathworks: Natick, MA, USA) [25].

### RT-PCR

A longer (50 min) enzymatic incubation and more complete trituration with glass pipettes were performed to obtain isolated cells. The digestive enzymes used were the same as described for cell cluster preparations. The resultant cell suspension was incubated in a ‘normal’ extracellular solution containing phycoerythrin-conjugated anti-mouse CD117 (c-Kit) antibody (PE-ACK2, eBioscience, San Diego, CA, USA) in 1/100 v/v for 10 min, and then centrifuged and rinsed with ‘normal’ solution three times. About 5–10 isolated smooth muscle cells and c-Kit-immunopositive cells were separately collected into sterile tubes, using patch pipettes (GC150-15, Harvard Apparatus, Kent, U.K.) with 10–20 µm tip diameter under a fluorescent microscope. The samples were kept at −80°C until use. The procedures for subsequent RT-PCR were the essentially the same as previously described [26].

The following PCR primers were used: 5-HT_2B_ (NM_008311, 1340-1443, amplicon  = 104 bp): 5′-GATCAACCCTGCCATGTACCA-3′ (+) and 5′-CGCCATCGTTTTCAGTGAGA-3′ (-); 5-HT_3A_ (NM_013561, 363-463, amplicon  = 101 bp): 5′-GACTCCTGAGGACTTCGACAATG-3′ (+) and 5′-ACTTCCCCACGTCCACAAACT-3′ (-); 5-HT_3B_ (NM_020274, 607-728, amplicon  = 122 bp): 5′-ACTCTTCTGGCACCATTAGAACC-3′ (+) and 5′-GAGGCTGCAGTTCTGGATATCA-3′ (-); 5-HT_4_ (NM_008313, 1100-1222, amplicon  = 123 bp): 5′-CTTTCCTCTGGCTTGGCTATATCA-3′ (+) and 5′-GTCTTTTGTAGCGCTCATCATCAC-3′ (-); c-Kit (Y00864, 2156-2256, amplicon  = 101 bp): 5′-GAGCCTTCCTGTGACAGTTCAAAT-3′ (+) and 5′-TCTATTCTTGCGGATCTCCTCTTG-3′ (-); glyceraldehyde-3-phosphate-dehydrogenase (GAPDH) (M32599, 730–833, amplicon  = 104 bp): 5′-CATGGCCTTCCGTGTTCCT-3′ (+) and 5′-CCTGCTTCACCACCTTCTTGA-3′ (-); CD68 (a marker for mast cells) (NM_009853, 574-830, amplicon  = 257 bp): 5′-CACCTGTCTCTCTCATTTCC-3′(+) and 5′-CTTAGAGAGAGCAGGTGAAG-3′ (-); Cma1 (a marker for macrophages) (NM_010780, 332–668, amplicon  = 337 bp): 5′-CGGGAAGGTCTATAACAGTCCTCC-3′ (+) and 5′-CTGGTGAAGTGTTTGCAGGCT-3′(-). The pair of primers for 5-HT_4_ was also designed to cover 5-HT_4A_, 5-HT_4B_, 5-HT_4E_, and 5-HT_4F_.

### Immunohistochemistry

Small segments (10 mm×20 mm) of smooth muscle layers (including the myenteric plexus) isolated from the mouse ileum, were fixed with 4% paraformaldehyde (4°C) for 30 min, and permeabilized with 0.1% triton X-100 and 5% BSA (bovine serum albumin, fraction V: Sigma) for 1 h. The tissue was double stained sequentially with anti-5-HT_3_ antibody [SR-3 (H-138) sc-28958: Santa Cruz Biotechnology, Santa Cruz, CA, USA] and anti-mouse CD117 (c-Kit) antibody (ACK4: Acris antibodies, Germany) in 100 mM PBS (phosphate buffered-saline solution) overnight. The PBS contained 1% BSA in order to block non-specific reactions. This was followed by incubation with secondary antibodies, Alexa Fluor 488-conjugated anti-rabbit IgG and Alexa Fluor 555-conjugated anti-rat IgG (Molecular Probes, Eugene, OR, USA) at a concentration of 15 µg/ml for 1 h. Double-stained small segments were mounted on a slide glass with an anti-fading agent (ProLong: Molecular Probes) and scanned using a confocal microscope (TCS-SP2: Leica Microsystems, Tokyo, Japan). Controls were prepared by omitting the primary antibodies. The reactivity was negligible in network-forming cells in the myenteric plexus (i.e. ICC). The antibody used for staining the 5-HT_3_ receptor (sc-28958: Santa Cruz Biotechnology) is a rabbit polyclonal antibody raised against amino acids 341–478 mapping at the C-terminus of 5-HT_3A_ of human origin, and is used in human, mouse and rat specimens.

### Solutions and drugs

The composition of the ‘normal’ extracellular solution used in [Ca^2+^]_i_ imaging and electrical recording was (in mM): NaCl, 125; KCl, 5.9; MgCl_2_ 1.2; CaCl_2_ 2.4; glucose 11; Tris-HEPES 11.8 (pH 7.4). Nifedipine, LY-278584, 2-methyl-5-HT (maleate salt), GR113808 and O-methyl-5-HT (hydrochloride) were purchased from Sigma. SK&F96365 was from Calbiochem (San Diego, CA, U.S.A.). Stock solutions of nifedipine were prepared by dissolving the drug in ethanol, while other drugs were dissolved in dimethyl sulfoxide (DMSO). The working concentrations of the solvents were less than 1%. In preliminary experiments, we observed that applications of this concentration of either solvent alone had little effect on [Ca^2+^]_i_ oscillation in cell cluster preparations. DMEM and other reagents for cell culture were purchased from Sigma.

### Statistics

Numerical data are expressed as mean±SD. Differences between means were evaluated by paired *t*-tests. The probability (*P*)<0.05 was taken as a statistically significant difference.

## Results

### 5-HT augments [Ca^2+^]_i_ activity in ICC

ICC generate pacemaker activity employing [Ca^2+^]_i_ oscillations [13], [14]. To examine the effect of 5-HT on [Ca^2+^]_i_ activity in ICC, we used cell cluster preparations (100–200 µm in diameter) obtained from the mouse ileum. This preparation contains ICC, smooth muscle cells and enteric neurons, and is considered to be an integrated model system to investigate gut pacemaker activity. Dihydropyridine (DHP) Ca^2+^ antagonists can selectively suppress [Ca^2+^]_i_ and contractile activities of smooth muscle cells by blocking L-type Ca^2+^ channels, while pacemaking [Ca^2+^]_i_ oscillations in ICC persist [19]. We therefore carried out [Ca^2+^]_i_ imaging in the presence of nifedipine.

A typical effect of 5-HT is shown in Fig. 1A. In the continuous presence of nifedipine (1 µM), spontaneous [Ca^2+^]_i_ oscillations were observed in a small limited area (near (x)) [control: Panels (a–b) in Fig. 1A]. Application of 5-HT (10 µM) enlarged the region showing spontaneous [Ca^2+^]_i_ oscillations [Panels (c–d)]. The time course of changes in [Ca^2+^]_i_ (F_t_/F_0_) is plotted in Fig. 1B. In control conditions (a), only the region of interest (ROI) (x) shows [Ca^2+^]_i_ oscillations with an amplitude of more than 1.2 (F_t_/F_0_), while such activity was negligible in ROI (y) and (z). After application of 5-HT, the area yielding spontaneous [Ca^2+^]_i_ oscillations enlarged, covering all three regions (x–z) synchronously. The peak amplitude in ROI (y) reached 80–90% of that in (x). On average, the area yielding [Ca^2+^]_i_ oscillations increased from 11.5±6.0 to 42.5±18.3% (n = 22), while the frequency was little affected (19.4±1.2 in normal vs. 19.5±1.7 cycles/min in the presence of 5-HT, n = 22) (Fig. 1C–E). Essentially similar effects of 5-HT were observed even in the presence of TTX as well as nifedipine (n = 5), suggesting that 5-HT augmented ICC pacemaker activity, but not through neural activity.

An interesting response was observed in preparations showing intermittent [Ca^2+^]_i_ oscillations. 5-HT (10 µM) caused the activity towards a continuous oscillation pattern, with a significant enlargement of the oscillation active area (n = 6) (Figure S2).

When [Ca^2+^]_i_ imaging was carried out in ileal cell cluster preparations from *W/W^v^* mice, which have few pacemaking interstitial cells in this part of the small intestine (Fig. 1F–G) [27]–[29], no [Ca^2+^]_i_ oscillation was observed irrespective of 5-HT application (in the presence of 1 µM nifedipine, n = 4) (Fig. 1H). This result confirms that ICC are responsible for 5-HT-mediated enhancement in normal mice.

### 5-HT receptor agonists and antagonists

Next, we assessed which receptors are responsible for 5-HT-mediated pacemaker [Ca^2+^]_i_ activity. In these experiments, we used preparations showing synchronous [Ca^2+^]_i_ oscillations (like the preparation shown in Fig. 1). Application of LY-278584 (10 µM), an antagonist for type 3 5-HT receptors (5-HT_3_) significantly decreased the amplitude of the oscillations, and the intervals between them often became irregular (middle traces in Fig. 2A–B). Almost complete recovery was achieved by additional application of 10 µM 2-methyl-5-HT (2-Me-5-HT), a selective agonist for 5-HT_3_ receptors (*F*_peak_ */F*_0_: 1.27±0.06 in control vs 1.25±0.06 with 2-Me-5-HT, n = 5) (the right trace in Fig. 2A). Also, in order to confirm the target cells, 2-Me-5-HT (10 µM) was applied in cell cluster preparations from *W/W^v^* mice, but no [Ca^2+^]_i_ oscillation was induced (n = 5).

O-methyl-5-HT (O-Me-5-HT) is a known non-selective 5-HT receptor agonist, but lacking affinity for 5-HT_3_ receptors. Application of O-Me-5-HT (10 µM) caused no recovery of spontaneous [Ca^2+^]_i_ activity in the presence of LY-278584 (10 µM)(n = 4, Fig. 2B). Essentially the same results as shown in Fig 2A–B were obtained in the presence of TTX (250 nM), indicating that there is no contribution of neural activity: Namely these drugs work through action potential-independent mechanisms.

In contrast to the effects of 5-HT_3_ antagonists, application of 10 µM GR113808, a selective antagonist for 5-HT_4_, had little effect on spontaneous [Ca^2+^]_i_ oscillations in ICC (*F*_peak_ */F*_0_: 1.25±0.06 in control vs 1.22±0.02, n = 5)(middle trace in Fig. 2C). Additional application of 10 µM LY-278584 again suppressed [Ca^2+^]_i_ activity (right trace in Fig. 2C). These pharmacological examinations, together suggest that pacemaker [Ca^2+^]_i_ activity in ICC is generated by endogenous 5-HT via 5-HT_3_ receptors.

Ca^2+^ influx sensitive to SK&F96365 plays an essential role in generating ICC pacemaker [Ca^2+^]_i_ activity, presumably co-ordinating with intracellular Ca^2+^ release channels [16], [17]. In order to assess whether this Ca^2+^ influx pathway contributes to the pacemaker activity mediated via 5-HT_3_ receptors, the effect of 2-Me-5-HT was examined in the presence of SK&F96365. Application of 40 µM SK&F96365 suppressed pacemaker [Ca^2+^]_i_ activity in ICC as observed previously [17], but subsequent application of 2-Me-5-HT caused no recovery (n = 5) (Fig. 2D). The responses of pacemaker [Ca^2+^]_i_ activity to the drugs relating to 5-HT receptors and Ca^2+^ influx are summarized in Fig. 2E.

### 5-HT_3_ receptors in ICC

**Figure 3:**
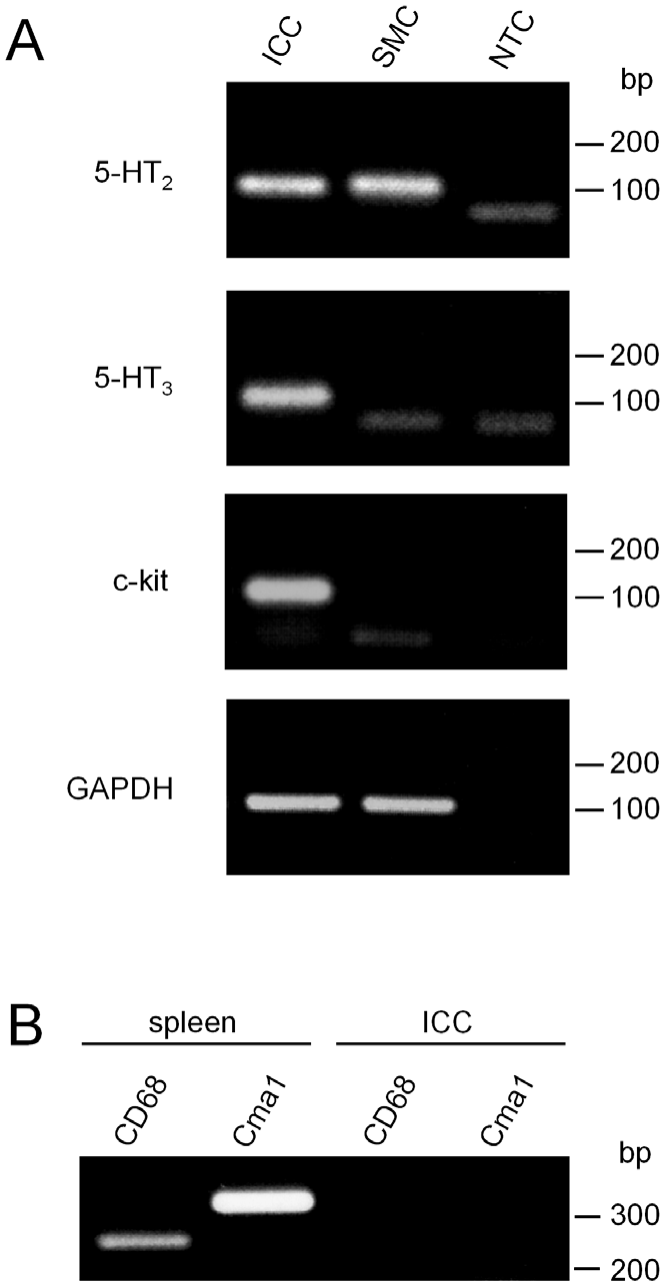
RT-PCR examinations for 5-HT receptors. A) RNA samples were obtained from isolated c-KIT-immunopositive cells (ICCs) or smooth muscle cells (SMC). NTC represents ‘no template control’. RT-PCR was performed for 5-HT_2B_, 5-HT_3A_, *c-kit* and GAPDH (an index of proper amplification). Numbers on the right of each gel indicate the size marker (bp). B) Examples of RT-PCR detection of mast cell markers (CD68 and Cma1) in spleen (35 cycles) and c-kit-immuopositive cells (50 cycles).

To confirm expression of 5-HT receptors in ileal ICC, we performed RT-PCR. After enzymatic dispersion, c-Kit immunopositive interstitial cells (equivalent to ICC) and smooth muscle cells were individually collected. A transcript for 5-HT_3A_ was detected only in ICC, while 5-HT_2B_ was detected in both ICC and smooth muscle cells (Fig. 3A). We also confirmed that neither CD68 nor Cma1 (mast cell markers) was detected in c-Kit-immunopositive interstitial cells (n = 4), unlike in spleen samples (n = 4) (Fig. 3B). In addition, transcripts for 5-HT_3B_ and 5-HT_4_ were detected in ICC, but not in smooth muscle cells (not shown).

**Figure 4:**
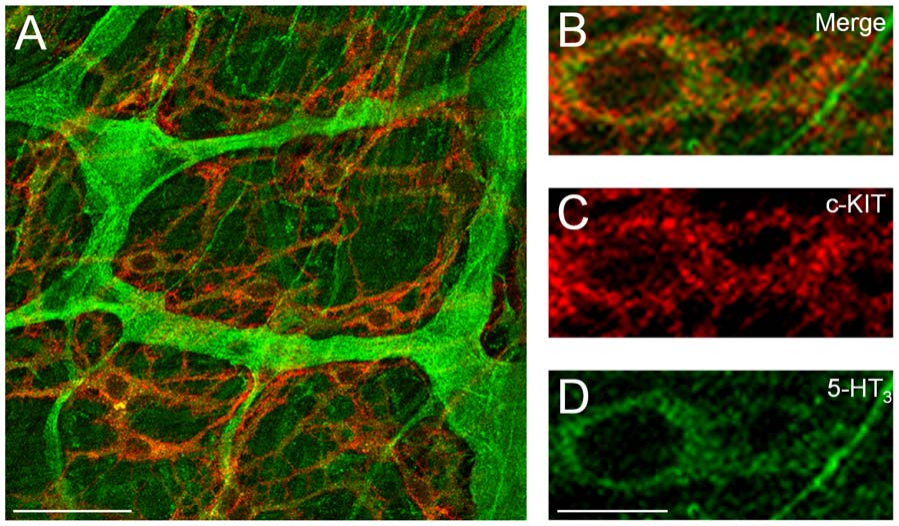
Immunohistochemistry for c-KIT and 5-HT_3_. Smooth muscle layer of the ileum including the myenteric plexus was double-labelled with anti-c-KIT antibody (ACK4, red) and anti-5-HT_3_ antibody (sc-28958, green). Left panel (A: merged image) shows myenteric neurons (plexus) (green) surrounded by network-forming cells expressing both c-KIT and 5-HT_3_. Bar, 50 µm. An ICC-like cell is shown expanded in right panels (B: merged image; C: c-KIT; D: 5-HT_3_). Bar, 10 µm. The network-like structure and large nucleus are known histological features of ICCs.

Immunohistochemistry was also performed. Double-labelled immunostaining with anti-c-Kit and anti-5-HT_3_ antibodies (Fig. 4) revealed that network-forming interstitial cells expressing both 5-HT_3_ and c-Kit (orange cells in left panel) exist near the myenteric plexus, which contains neurons with only 5-HT_3_ immunoreactivity (green). In addition, these network-forming cells have large nuclei (a single cell is shown expanded in the right panels). The network-like structure and large nuclei are known features of ICC [2], [6].

### Augmentation of electrical activity

In order to confirm the excitatory effect of 5-HT on ICC pacemaker activity, we next measured electrical activity. Isolated musclature of the mouse ileum was placed on an 8×8 microelectrode array (MEA) with a polar distance of 150 µm, and field potentials of a ∼1 mm^2^ area were simultaneously monitored through a multi-channel amplifier and recording system (See Materials and Methods). To suppress smooth muscle and neural activities, extracellular solution contained nifedipine (1 µM)) and TTX (250 nM), respectively.

**Figure 5:**
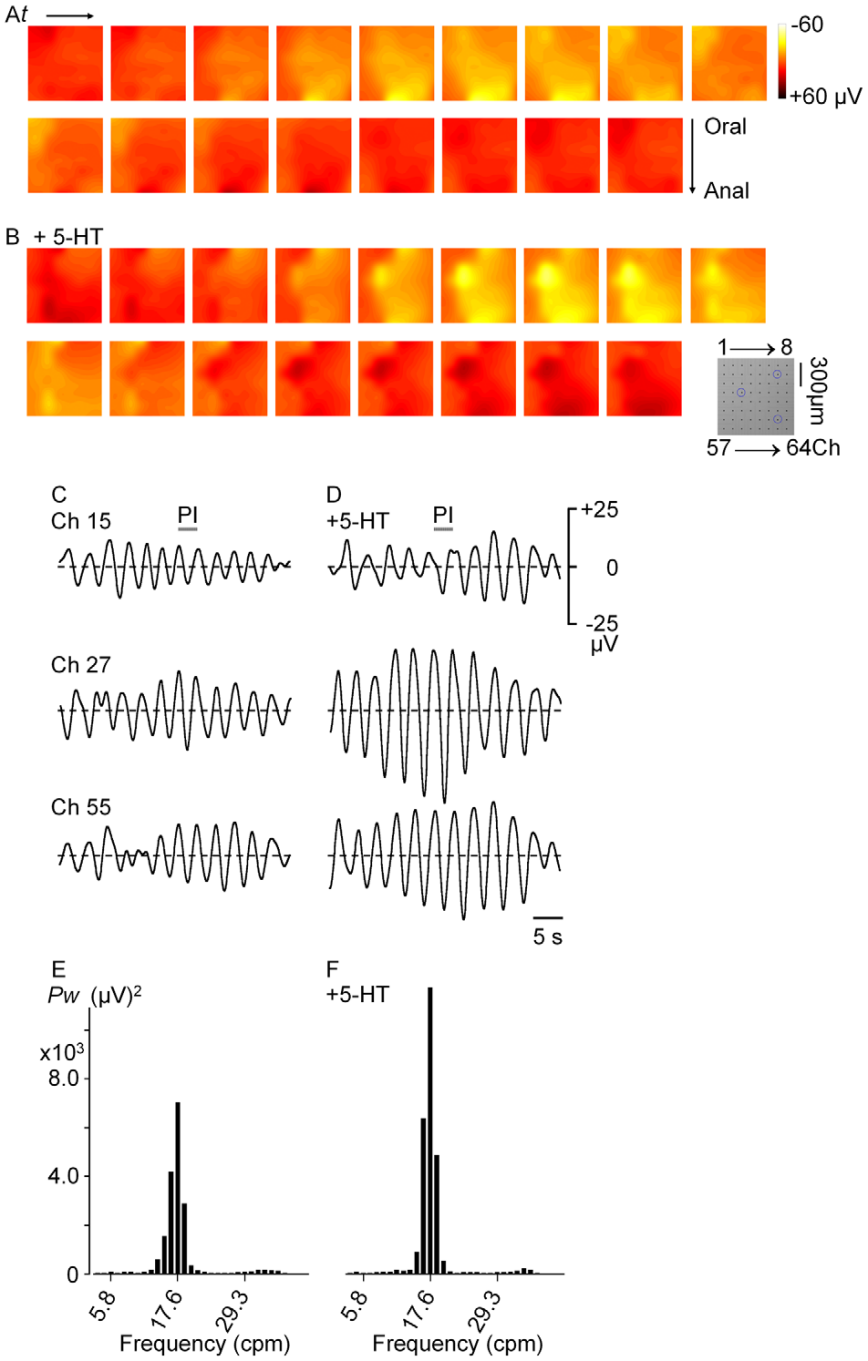
MEA measurements of ICC electrical activity in the presence of nifedipine (1 µM) and TTX (250 nM). A-B) Field potential images constructed at 200 ms. The top and bottom of the images correspond to the oral and anal ends of the preparation, respectively. C-D) Representative field potential recordings at three channels in the same preparation. The dotted lines indicate the period of the potential images (PI) acquired. E-F) Power spectra constructed from field potential recordings at all 64 channels. A, C and E: control; B, D and F: during application of 5-HT.

A potential map was constructed by using the simultaneous recordings at 64 channels and spline interpolation (Fig. 5A–B). In this preparation, ICC electrical activity propagated from the left bottom to the right top in normal solution (A), and application of 5-HT (10 µM) potentiated ICC electrical activity (B). Representative field potentials of three channels (Fig. 5C–D) show ICC electrical activities for a long duration. 5-HT significantly increased the amplitude of the field potentials, but did not alter the frequency (Video S3 and Video S4). Also, power spectra over the recording area were constructed from field potentials of all 64 channels for approximately 40 s. This analysis shows a marked increase in the spectral power without shifting the peak frequency.

**Figure 6:**
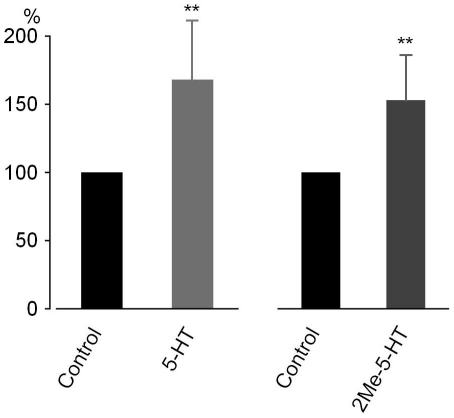
Potentiation of ICC electrical activity with 5-HT (10 µM) and 2-Me-5-HT (10 µM). All measurements were carried out in the presence of nifedipine (1 µM) and TTX (250 nM).

The effect of 2-Me-5-HT (10 µM), a 5-HT_3_ receptor agonist (Figure S3), on ICC electrical activity was also examined. Essentially similar enhancement was observed. Graphs in Fig. 6 summarise the enhancement of 5-HT and 2-Me-5-HT on ICC electrical activity. The spectral power between 9.4-27.0 cpm (*Pw*_9.4-27.0cpm_) was used to evaluate the ICC electrical activity, based on a comparison between wild-type and *W/W^v^* mice [23]. In the control of normal ileum, *Pw*_9.4–27.0cpm_ (from 64 channels) ranged from 1.0 to 19.0×10^−3^ µV^2^ (n = 24). Application of 5-HT increased *Pw*_9.4–27.0cpm_ to 168±45% (*P*<0.01, n = 12), and 2-Me-5-HT increased it to 153±35% (*P*<0.01, n = 12).

## Discussion

ICC act as gut pacemaker cells. Moreover, fairly recent studies suggest that these cells also coordinate peristaltic movements through their network-forming structure [30]. In the light of the important roles of ICC, any hormones and neurotransmitters that modulate ICC activity are considered to have a significant influence upon gut motility. The present finding that 5-HT augments ICC activity implies that this signalling molecule in particular plays a crucial role in regulating gut motility, because the gut contains a majority of 5-HT in the body [8], [11].

The fact that 2-Me-5-HT causes similar effects to 5-HT implies that 5-HT_3_ receptors are responsible for the 5-HT-mediated enhancement of ICC Ca^2+^ activity. Lines of studies have suggested that the primary pacemaking activity is the spontaneous [Ca^2+^]_i_ oscillations in ICC [13], [15], [31]. Namely, [Ca^2+^]_i_ oscillations in ICC periodically activate plasmalemmal Ca^2+^-activated ion channels: Ca^2+^-activated Cl^−^ channels [32]–[35], and/or Ca^2+^-activated non-selective cation channels [36]–[38]. Ca^2+^ release from the intracellular Ca^2+^ stores, presumably endoplasmic reticulum (ER), appears to be the major Ca^2+^ source of [Ca^2+^]_i_ oscillations in ICC, while Ca^2+^ influx from the extracellular space is required to maintain this [Ca^2+^]_i_ activity (periodic Ca^2+^ release) [13], although co-ordinating mechanisms for these Ca^2+^ pathways are not yet understood. Since 5-HT_3_ receptors are ligand-gated non-selective cation channels, permeable to Ca^2+^ [39], [40], this channel is likely to act as a Ca^2+^ influx pathway to enhance pacemaker activity in ICC (Figure S4). This notion is supported by the fact that SK&F96365, which is known to block a broad range of Ca^2+^-permeable non-selective cation channels [41]–[43], terminates ICC pacemaker Ca^2+^ activity even in the presence of 2-Me-5-HT (Fig. 2D). Also, ICC is known to express DHP-insensitive voltage-gated Ca^2+^-permeable channels [44], [45]. This transmembrane Ca^2+^ pathway may simultaneously contribute to the enhancement of ICC [Ca^2+^]_i_ activity.

Five subunits (i.e. 5-HT_3A–E_) are known to form the 5-HT_3_ receptor complex, and changes in the composition alter Ca^2+^-permeability of this channel [46]. In future studies, it would be of interest to elucidate the composition of 5-HT_3A-E_ receptor subtypes and how 5-HT_3_ receptors are coupled to Ca^2+^ release channels in intracellular Ca^2+^ stores to generate pacemaker [Ca^2+^]_i_ activity. Polymorphism of these 5-HT_3_ receptor subunit genes seems likely to affect gut motility by modulating ICC as well as neuronal activities [12], and underlies some functional disorders [47]. Furthermore, RT-PCR examinations detected transcripts of other 5-HT receptor genes in ICC i.e. 5-HT_2_ and 5-HT_4_ receptor genes [48], [49]. Recent studies have shown that 5-HT_2B_ receptor antagonists reduced proliferation of cultured ICC, and that the small intestine of mice lacking 5-HT_2B_ receptors contains less ICC in the myenteric and deep muscular plexuses, although intestinal transit is not significantly slowed [50], [51]. On the other hand, 5-HT_4_ receptor antagonists impair the regeneration of enteric neurons after surgical operation and their development in gut-like organs derived from mouse embryonic stem cells, with indistinguishable changes in the ICC network [21], [52]. It is likely that 5-HT causes numerous effects via these different 5-HT receptors, depending on cell type, location of the gut, and the stage of development and aging.

The scenario for 5-HT augmentation of ICC activity is possibly modified by the roles of adjacent cells in the actual gut. In the present study, we applied nifedipine and TTX to clearly demonstrate the effect of 5-HT on ICC; however, smooth muscle cells and enteric neurones suppressed by these inhibitors may also be involved, because coordinated actions of these cells produce gut motility [4]. For example, as seen in Fig. 5, ICC pacemaker activity propagates on the luminal plane. Indeed, electric conduction of gut pacemaker activity along the musculature can be detected magnetically [53]. It is thought that ICC and smooth muscle cells are electrically connected [54]. Therefore, under normal conditions (without DHP Ca^2+^ antagonists), in addition to network-forming processes in ICC, the smooth muscle bundle conducts a part of electric current generated by a group of ICC, and amplifies pacemaker activity in adjacent ICC, because it is thought that ICC possess a mechanism to transform depolarisation in the plasma membrane into activation of [Ca^2+^]_i_ oscillations for pacemaking [55], [56]. Also, some populations of enteric neurones may release activators for ICC pacemaker activity in response to 5-HT. In the myenteric plexus, serotonergic neurones are involved in descending contraction [9], [10], and it is known that ICC express numerous receptors for excitatory neurotransmission, e.g. purinoceptors, neurokinin and acetylcholine receptors [57]–[60]. These molecules may access ICC to activate in parallel.

The present finding on augmentation of ICC activity via 5-HT_3_ receptors implies pharmacological interventions on gut motility disorders. For example, irritable bowel syndrome, classified into two types, i.e. diarrhoea- and constipation-dominant IBS (d-IBS and c-IBS), is known to involve 5-HT-related mechanisms along with infectious and inflammatory changes. Excess 5-HT due to impairment of reuptake transport is ascribed to some populations of d-IBS [61]–[63]. Also, antineoplastic drugs, e.g. cisplatinum, stimulate 5-HT release [64]–[66]. In such cases, it is rational to suppress ICC pacemaker activity in addition to nervous activities by blocking 5-HT_3_ receptors. It is speculated that stimulation of 5-HT_3_ receptors in enteric neurons and ICC synergically facilitates gut contractility and afferent neural activity toward the brain. Thereby, 5-HT_3_ receptors in the gut may contribute to the gut-brain axis. As seen in murine ileal ICC, we have also observed that 5-HT_3_ receptor agonists potentiate, while antagonists suppress both Ca^2+^ and electric pacemaker activities in the murine stomach in preliminary experiments. Although extensive studies are required in model animals and humans, 5-HT is likely to enhance ICC pacemaker activity throughout the gastrointestinal tract.

Similar regulatory mechanisms may underlie other peripheral spontaneous activities. Evidence is being accumulated that ICC-like interstitial cells ubiquitously exist in many organs and tissues that are effectors of the autonomic nervous system, such as the ureter, urinary bladder, urethra, uterus, lymph ducts, veins, etc, suggesting their possible contribution to spontaneous activity [67]–[71]. Also, in some ICC-like cells, spontaneous [Ca^2+^]_i_ and electric activities have already been demonstrated. In the light of regulatory mechanisms of ICC and ICC-like cells, investigating functional disorders related to a wide range of peripheral spontaneous rhythmicity, e.g. irritable bladder, is merited.

In summary, the present study has shown 5-HT augmentation of ICC pacemaker activity via 5-HT_3_ receptors. Since 5-HT_3_ receptors are Ca^2+^-permeable nonselective cation channels, this effect on ICC activity is presumably through enhancement of Ca^2+^ influx from the extracellular space, through itself and simultaneous activation of voltage-gated Ca^2+^-permeable channels. ICC appear to be a promising target in functional motility disorders in the gut.

## Supporting Information

Figure S1 An example of 5-HT-augmentation of ICC pacemaker [Ca^2+^]_i_ oscillations in an ileal musculature preparation. Ileal musculature segments (∼5 mm×20 mm) containing the myenteric plexus, the same preparation used in 8×8 MEA measurements, were loaded with Fluo-3AM. Fluo-3 emission light images were continuously monitored the same as cell cluster experiments. The extracellular solution contained 1 µM nifedipine and 250 nM TTX. A) A control fluorescent image. B and C) Series of ratio images of a [Ca^2+^]_i_ oscillation cycle in control and in the presence of 5-HT (10 µM), respectively. Each image was acquired at 300 ms intervals. Video S1 and Video S2 correspond to ratio images shown in B and C, respectively. Note that green represents the *F*_t_ */F*_0_ ratio of ∼1 in cell cluster preparations (Fig. 1A) to display the size of the preparation, while black represents the ratio of ∼1 in musculature preparations, because the size of preparations were larger than the frame of the image. After 5-HT application the active area markedly increased.
(TIF)

Figure S2 Regular occurrence of pacemaker [Ca^2+^]_i_ oscillations after application of 5-HT. A) Ca^2+^ images acquired from a cell cluster preparation in control (a–c) and 5 min after 5-HT (10 µM) application (d, e). This preparation showed intermittent [Ca^2+^]_i_ oscillations in control condition. B) Time course of pacemaker [Ca^2+^]_i_ activity recorded in the square (x) indicated in A: control (left) and 5 min after application of 10 µM 5-HT (right). Dotted lines correspond to the times when images (a–e) were acquired. C–E) Bar graphs showing changes in the peak amplitude (C), frequency (D), and active area (E) of spontaneous pacemaker [Ca^2+^]_i_ activity (Mean ± S.D., n = 6).
(TIF)

Figure S3 An example of the effect of 2-Me-5-HT (10 µM) on field potentials. Field potential images in control (A) and during application of 2-Me-5-HT (B) are displayed at 200 ms intervals. Note, 2-Me-5-HT-enhanced ICC electrical activity, as seen during 5-HT application.
(TIF)

Figure S4 Possible underlying mechanisms for 5-HT-enhancement of gut pacemaker activity and contractility. It is thought that intracellular Ca^2+^ release channels, i.e. ryanodine receptors (RyR) and Ins*P*_3_ receptors (IP_3_R) in ICC are periodically activated by the support of a Ca^2+^ influx pathway across the plasma membrane, although mechanisms underlying the coordinated actions of intracellular and plasma membrane ion channels are not yet known. 5-HT augments ICC [Ca^2+^]_i_ oscillations presumably (1) by facilitating Ca^2+^ influx via 5-HT_3_ receptors, and (2) simultaneous activation of voltage-gated Ca^2+^-permeable insensitive to DHP Ca^2+^ antagonists (DHP(-)VGCC). In ICC, [Ca^2+^]_i_ oscillations periodically activate Ca^2+^-activated ion channels in the plasmamembrane, i.e. Ca^2+^-activated Cl^−^ channels (Cl_Ca_) and/or Ca^2+^-activated nonselective cation channels (NSCC_Ca_), and thereby generate electric pacemaker activity. In smooth muscle (SM) cells, conducted pacemaker activity via gap junction (GJ) channels activates DHP-sensitive voltage-gated Ca^2+^ channels (DHP(+)VGCC), i.e. L-type Ca^2+^ channels, causing periodic contraction. In the present study, to differentiate ICC activity, all experiments were carried out in the presence of a DHP Ca^2+^ antagonist, nifedipine.
(TIF)

Video S1 [Ca^2+^]_i_ oscillations measured in a musculature preparation in normal condition, corresponding to Figure S1B.
(MPG)

Video S2 [Ca^2+^]_i_ oscillations measured in a musculature preparation in the presence of 5-HT (10 µM), corresponding to Figure S1C.
(MPG)

Video S3 Field potential oscillations acquired under a control condition, corresponding to Figure 5A. An 8×8 microelectrode array with a polar distance of 150 µm was used. The recording area was ∼1 mm^2^.
(MPG)

Video S4 Field potential oscillations acquired 5 min after application of 10 µM 5-HT corresponding to Figure 5B.
(MPG)
