# Supplementary figures and images for: Serotonin Augments Gut Pacemaker Activity via 5-HT3 Receptors
Source: PLoS One. 2011 Sep 15;6(9):e24928. doi: 10.1371/journal.pone.0024928 (PMC3174222; doi:10.1371/journal.pone.0024928)

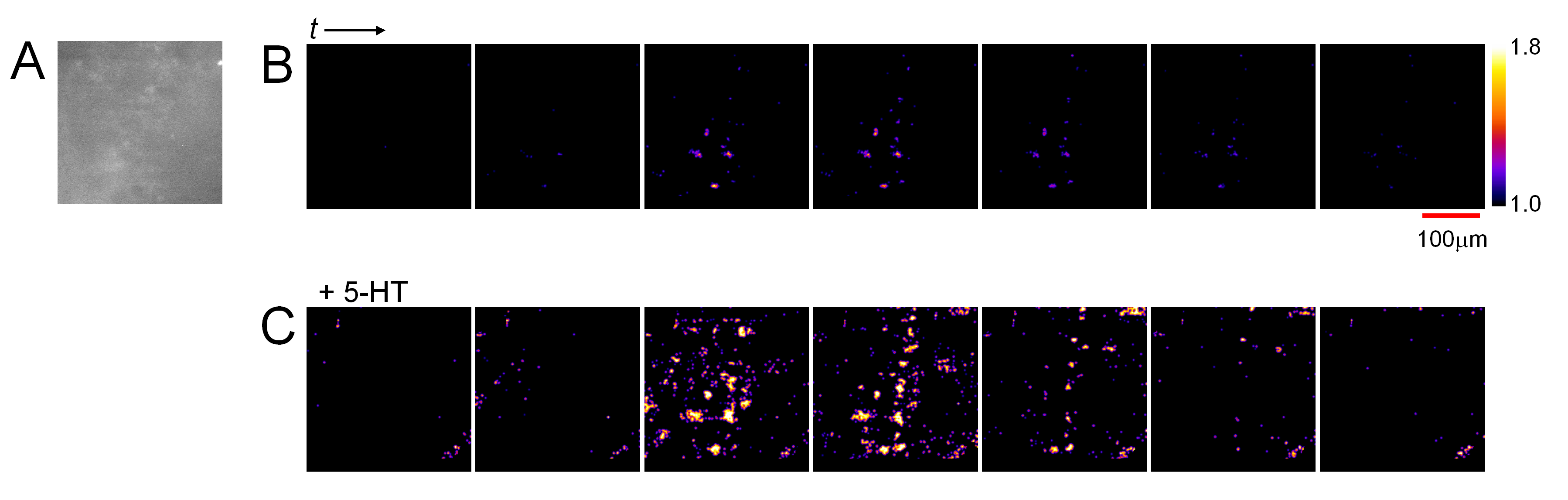

Supplement: Figure S1 — An example of 5-HT-augmentation of ICC pacemaker [Ca2+]i oscillations in an ileal musculature preparation. Ileal musculature segments (∼5 mm×20 mm) containing the myenteric plexus, the same preparation used in 8×8 MEA measurements, were loaded with Fluo-3AM. Fluo-3 emission light images were continuously monitored the same as cell cluster experiments. The extracellular solution contained 1 µM nifedipine and 250 nM TTX. A) A control fluorescent image. B and C) Series of ratio images of a [Ca2+]i oscillation cycle in control and in the presence of 5-HT (10 µM), respectively. Each image was acquired at 300 ms intervals. Video S1 and Video S2 correspond to ratio images shown in B and C, respectively. Note that green represents the F t /F 0 ratio of ∼1 in cell cluster preparations (Fig. 1A) to display the size of the preparation, while black represents the ratio of ∼1 in musculature preparations, because the size of preparations were larger than the frame of the image. After 5-HT application the active area markedly increased. (TIF) [file pone.0024928.s001.tif]

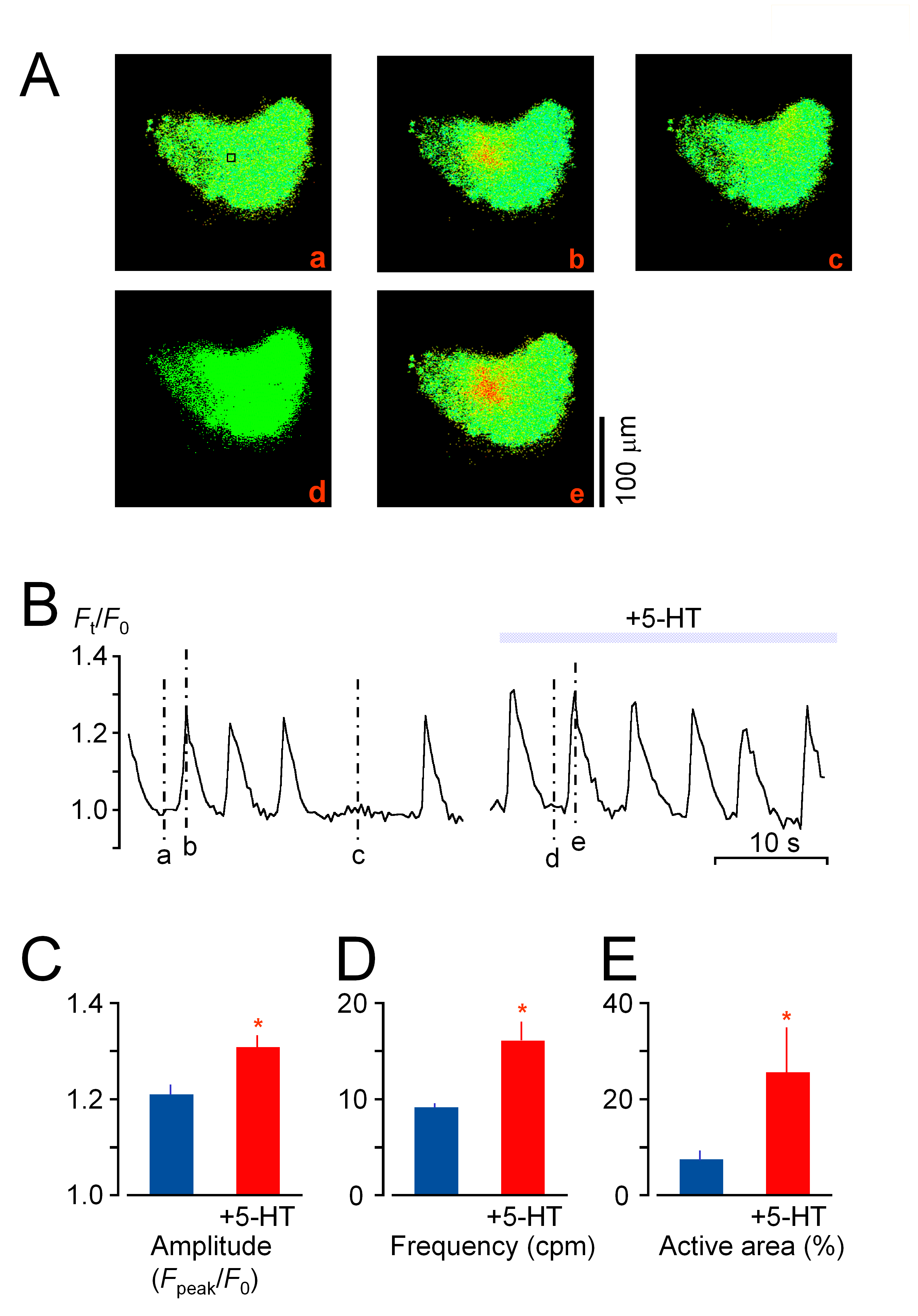

Supplement: Figure S2 — Regular occurrence of pacemaker [Ca2+]i oscillations after application of 5-HT. A) Ca2+ images acquired from a cell cluster preparation in control (a–c) and 5 min after 5-HT (10 µM) application (d, e). This preparation showed intermittent [Ca2+]i oscillations in control condition. B) Time course of pacemaker [Ca2+]i activity recorded in the square (x) indicated in A: control (left) and 5 min after application of 10 µM 5-HT (right). Dotted lines correspond to the times when images (a–e) were acquired. C–E) Bar graphs showing changes in the peak amplitude (C), frequency (D), and active area (E) of spontaneous pacemaker [Ca2+]i activity (Mean ± S.D., n = 6). (TIF) [file pone.0024928.s002.tif]

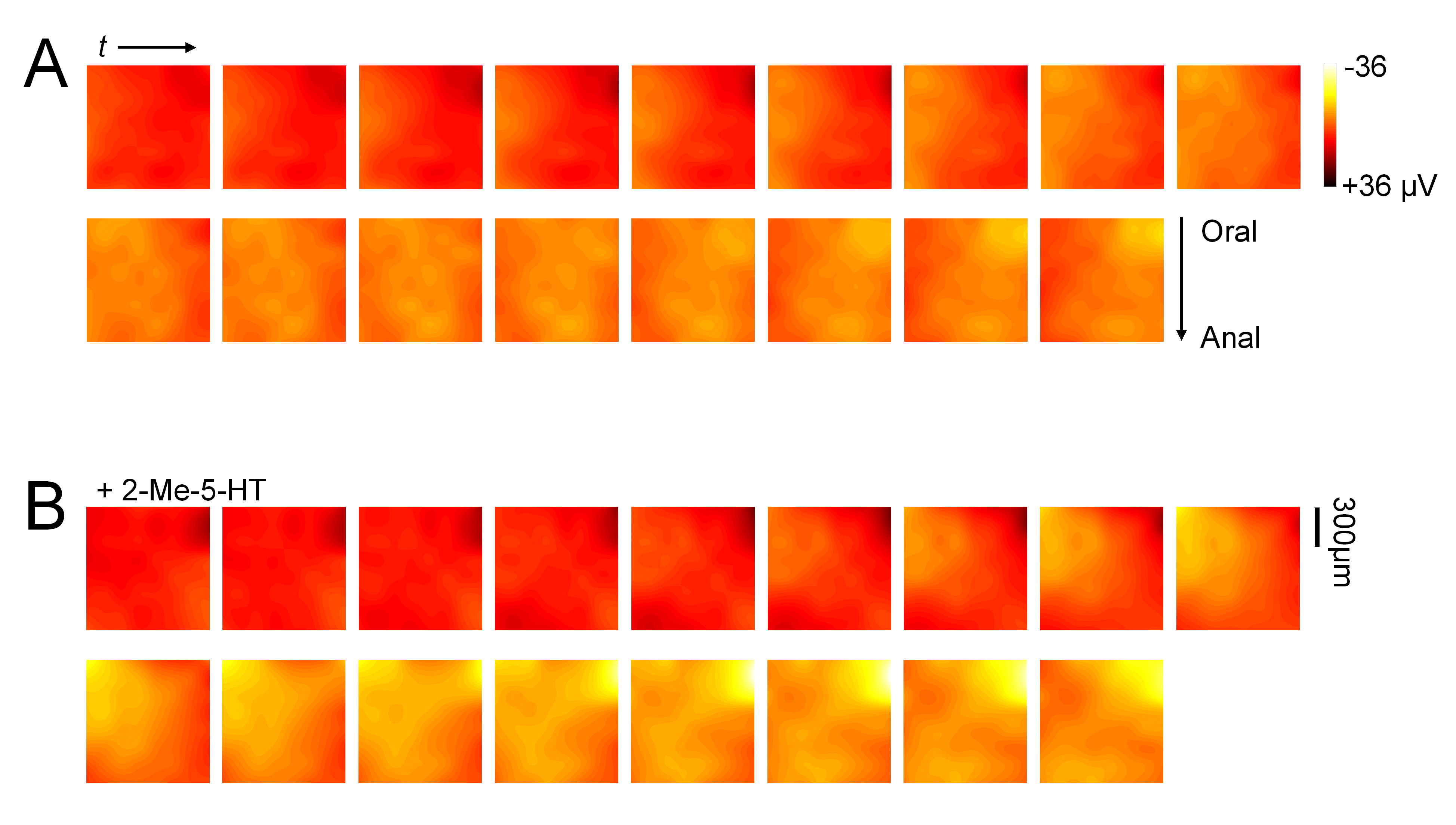

Supplement: Figure S3 — An example of the effect of 2-Me-5-HT (10 µM) on field potentials. Field potential images in control (A) and during application of 2-Me-5-HT (B) are displayed at 200 ms intervals. Note, 2-Me-5-HT-enhanced ICC electrical activity, as seen during 5-HT application. (TIF) [file pone.0024928.s003.tif]

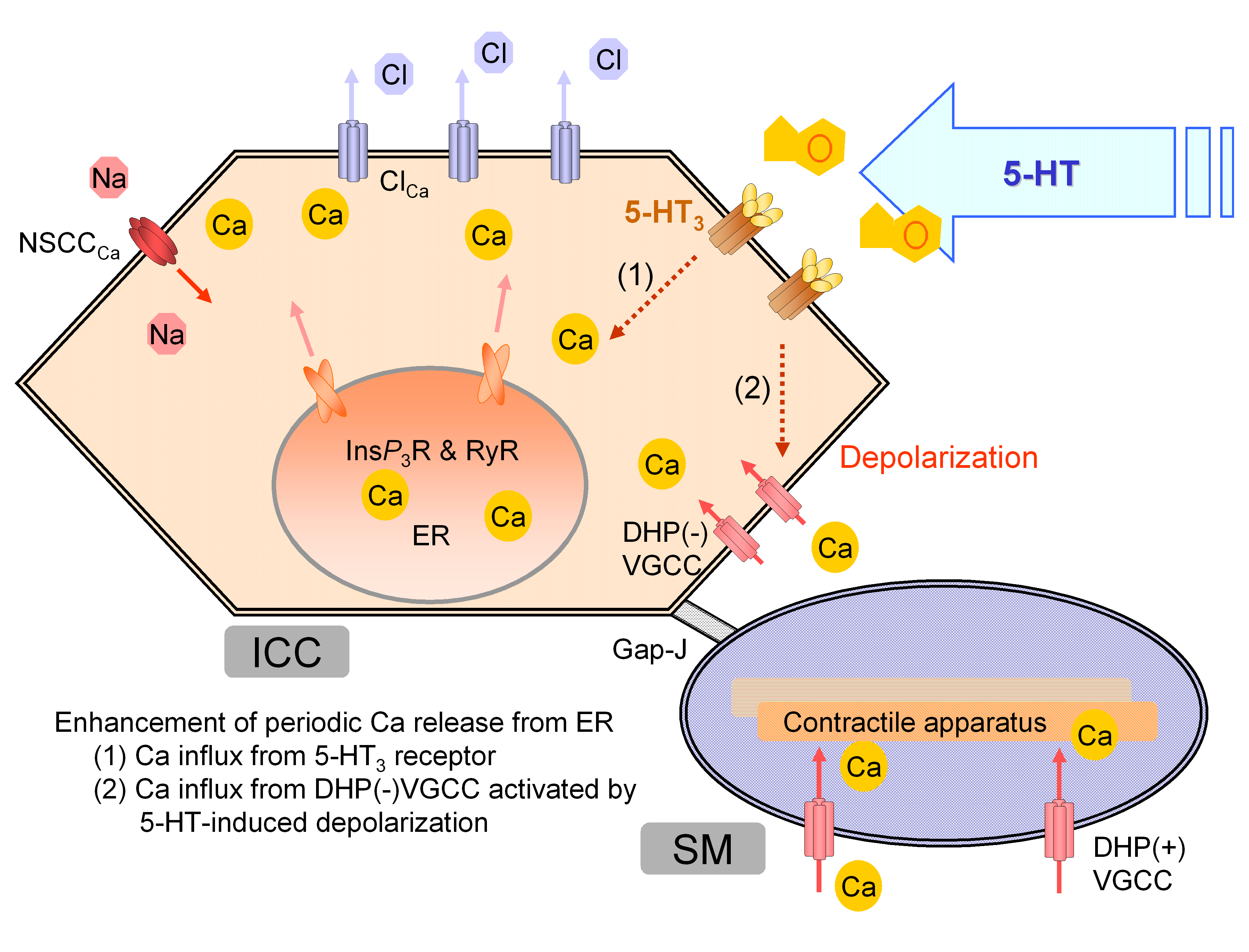

Supplement: Figure S4 — Possible underlying mechanisms for 5-HT-enhancement of gut pacemaker activity and contractility. It is thought that intracellular Ca2+ release channels, i.e. ryanodine receptors (RyR) and InsP 3 receptors (IP3R) in ICC are periodically activated by the support of a Ca2+ influx pathway across the plasma membrane, although mechanisms underlying the coordinated actions of intracellular and plasma membrane ion channels are not yet known. 5-HT augments ICC [Ca2+]i oscillations presumably (1) by facilitating Ca2+ influx via 5-HT3 receptors, and (2) simultaneous activation of voltage-gated Ca2+-permeable insensitive to DHP Ca2+ antagonists (DHP(-)VGCC). In ICC, [Ca2+]i oscillations periodically activate Ca2+-activated ion channels in the plasmamembrane, i.e. Ca2+-activated Cl− channels (ClCa) and/or Ca2+-activated nonselective cation channels (NSCCCa), and thereby generate electric pacemaker activity. In smooth muscle (SM) cells, conducted pacemaker activity via gap junction (GJ) channels activates DHP-sensitive voltage-gated Ca2+ channels (DHP(+)VGCC), i.e. L-type Ca2+ channels, causing periodic contraction. In the present study, to differentiate ICC activity, all experiments were carried out in the presence of a DHP Ca2+ antagonist, nifedipine. (TIF) [file pone.0024928.s004.tif]
